# “Still a Cancer Patient”—Associations of Cancer Identity With Patient-Reported Outcomes and Health Care Use Among Cancer Survivors

**DOI:** 10.1093/jncics/pky031

**Published:** 2018-07-05

**Authors:** Melissa S Y Thong, Eva-Maria Wolschon, Lena Koch-Gallenkamp, Annika Waldmann, Mechthild Waldeyer-Sauerland, Ron Pritzkuleit, Heike Bertram, Hiltraud Kajüter, Andrea Eberle, Bernd Holleczek, Sylke R Zeissig, Hermann Brenner, Volker Arndt

**Affiliations:** 1 Unit of Cancer Survivorship, Division of Clinical Epidemiology and Aging Research, German Cancer Research Center (DKFZ), Heidelberg, Germany; 2 Institute for Social Medicine and Epidemiology, University of Lübeck, Lübeck, Germany; 3 Division of Clinical Epidemiology and Aging Research, DKFZ, Heidelberg, Germany; 4 Hamburg Cancer Registry, Ministry of Health and Consumer Protection, Hamburg, Germany; 5 Cancer Registry of Schleswig-Holstein, Lübeck, Germany; 6 Cancer Registry of North Rhine-Westphalia, Bochum, Germany; 7 Bremen Cancer Registry, Leibniz Institute for Prevention Research and Epidemiology - BIPS, Bremen, Germany; 8 Saarland Cancer Registry, Saarbrücken, Germany; 9 Cancer Registry of Rhineland-Palatinate, Mainz, Germany; 10 Division of Preventive Oncology, DKFZ and National Center for Tumor Diseases (NCT), Heidelberg, Germany; 11 German Cancer Consortium (DKTK), DKFZ, Heidelberg, Germany

## Abstract

**Background:**

The concept of cancer identity is gaining attention as more individuals are living with cancer as a chronic illness. Research is limited, and results suggest that a self-identity as “cancer patient” rather than a “cancer survivor” is associated with depression and lower health-related quality of life (HRQL). We aimed to identify factors associated with patient identity and investigate the associations between patient identity and treatment, health care use, psychosocial distress, and HRQL.

**Methods:**

We used data from the population-based CAncEr Survivorship: A multi-Regional (CAESAR) study. Breast, colorectal, and prostate cancer survivors diagnosed during 1994–2004 completed a postal survey on patient identity, HRQL, psychological distress, and health care use in 2009–2011. We calculated odds ratios and the 95% confidence interval of having a patient identity. Analyses were adjusted for age, sex, education, and cancer stage, where appropriate.

**Results:**

Of the 6057 respondents, colorectal cancer survivors (25%) were least likely to consider themselves patients, and prostate cancer survivors (36%) the most likely. Being male, younger age, comorbidity, higher cancer stage, and disease recurrence were associated with patient identity. Treatment was associated with patient identity, except among female colorectal cancer survivors. Having a patient identity was associated with higher health care use within the past 12 months. Survivors who still consider themselves patients were more likely to be depressed and reported significantly lower HRQL.

**Conclusions:**

A significant proportion of cancer survivors still consider themselves patients five to 15 years postdiagnosis. Sensitivity to individuals’ self-identity should be considered when exploring their cancer experience.

Cancer is no longer deemed a death sentence. In 2012, more than 32 million individuals worldwide were still living five years postdiagnosis (1). This number is projected to increase significantly in the coming decades, mainly due to the combined factors of an aging population, increased cancer incidence, and improvements in detection and treatments (2). In the United States, the number of individuals living with a history of cancer is estimated to increase from approximately 14 million in 2012 to 18 million by 2022, of whom over 60% will have survived five years, 40% 10 years, and 15% 20 or more years (3). Similar trends were observed in Germany in 2013 with a five- and 10-year relative survival rates of approximately 60% and approximately 40%, respectively (4).

As more individuals now live with cancer as a chronic illness or consider themselves “cured,” concepts such as cancer survivorship and cancer identity are gaining attention (5). Individuals are more likely to describe themselves as a “cancer survivor,” with its positive connotation of empowerment, replacing the traditional labels of “cancer victim” or “cancer patient” (6). Nevertheless, survivors in long-term remission but still dealing with the emotional or physical consequences may identify themselves as patients (6,7).

The prevalence of survivors who do not identify themselves as a survivor varies according to cancer types (breast 22%, prostate 69%, and gynecological 45%) (8). Among long-term prostate cancer survivors, 9% still consider themselves patients (9). The majority of long-term colorectal cancer survivors perceive themselves as survivors (55%) or persons who had had cancer (39%), and only 3% perceive themselves as cancer patients or victims (10).

Most research on cancer identity has focused on identifying factors associated with the survivor identity. Individuals with a survivor identity tend to be older (10), optimistic (5), have a positive affect (9), have adopted active coping strategies (5), adjusted better following cancer (11), reported more benefit finding and acceptance after cancer (10), and have greater life satisfaction (10). Perceiving a lower recurrence risk was associated with the survivor identity (12). In qualitative studies, fear of recurrence was a factor for not embracing the survivor identity (13,14). Factors associated with a patient identity included shorter time since diagnosis (15) and symptom burden (5). Individuals with a patient identity were more likely to be depressed (5) and reported lower health-related quality of life (HRQL) (11). Nevertheless, research on the prevalence, influencing factors, and outcomes of cancer identity is limited. Studies are often qualitative in design, are not population based, are focused mainly on breast and prostate cancers, and tend to have small samples (8).

The patient identity could have economic implications as individuals who still perceive themselves as patients could have higher health care use due to the long-term/late consequences of their disease and treatment. Previous studies have suggested that individuals’ perceptions of their chronic illness are associated with more visits to the emergency department (16) and greater use of primary health care (17). However, we found no published studies that investigated the association between cancer identity and health care use.

In this population-based study of 5 to 15 years–postdiagnosis cancer survivors, we aimed to investigate the proportion of survivors who still consider themselves patients and identify factors associated with cancer identity. Furthermore, we investigated the associations between cancer identity and health care use, psychosocial distress, and HRQL.

## Methods

### Setting and Participants

The population-based CAncEr Survivorship–A multi-Regional (CAESAR) study aimed to describe the long-term HRQL of breast, colorectal, and prostate cancer survivors. The study was conducted by the German Cancer Research Center (Deutsches Krebsforschungszentrum [DKFZ]) in collaboration with six population-based cancer registries in Germany (Bremen, Hamburg, North Rhine-Westphalia, Rhineland-Palatinate, Saarland, and Schleswig-Holstein). Cancer survivors diagnosed during 1994–2004 and registered in the participating cancer registries who were age 20–75 years at diagnosis were eligible.

The ethics committee of the University of Heidelberg and the local ethics committees of the participating cancer registries approved the study. All participants provided written informed consent.

### Data Collection

Data collection was conducted from 2009 to 2011 by postal questionnaire. Depending on the cancer registry, the participants were contacted directly by the cancer registry/regional study center (Hamburg, Schleswig-Holstein) or via the treating/study physician (Bremen, Rhineland-Palatinate, North Rhine-Westphalia, Saarland).

#### Cancer Identity

One question assessed patient identity: “Do you still see yourself as a cancer patient?” Survivors answered with either a “yes” or a “no.”

#### Health Care Use

Survivors completed one item assessing whether cancer treatment or aftercare had been completed. Six items assessed cancer-related visits in the past 12 months to 1) a general practitioner (GP), 2) a medical specialist (MS; eg, oncologist or psychologist), 3) a nonmedical practitioner (NP; eg, complementary medicine), 4) a hospital for acute care (AH), 5) a university hospital (UH), or 6) a rehabilitation hospital (RH). Items were answered with either a “yes” or “no.”

#### Psychosocial Distress

##### Level of strain

One item assessed the level of strain due to cancer that survivors are currently experiencing. Answers ranged from 1 (“none”) to 4 (“very”).

##### Questionnaire on Stress in Cancer Survivors

The 10-item Questionnaire on Stress in Cancer Survivors (QSC-R10) is a validated instrument assessing distress experienced by cancer survivors in daily life (18). Item scores ranged from 0 (“not applicable”) to 5 (“a very serious problem”), yielding a maximum score of 50. A cutoff score of greater than 14 was indicative of psychosocial distress (18).

##### Geriatric Depression Scale

The 15 items of the validated Geriatric Depression Scale (GDS) were answered with either a “yes” or a “no” (19). Out of a maximum score of 15, 5–10 suggests depression (“subclinical”) and 11 or higher indicates depression.

##### Fear of Progression Questionnaire

The Fear of Progression Questionnaire (FoP-Q-SF) is a validated reliable instrument assessing fear of recurrence (FoR) in chronically ill persons (20). Items are scored on frequency of experience of fear/worry: 1 (“never”) to 5 (“very often”). Moderate FoR is indicated with a cutoff score of 4 or higher on at least 50% of items and high FoR is indicated with a cutoff score of 4 or higher on at least 75% of items (21).

#### HRQL

HRQL was assessed with the European Organization for Research and Treatment of Cancer Quality of Life Core-30 (EORTC-QLQ-C30) questionnaire (22). This 30-item questionnaire consists of five functioning scales, a global health status/quality of life scale, and nine single items/scales on symptoms and financial impact. Item scores ranged from 1 (“not at all”) to 4 (“very much”), with the exception of the global health status scale, which is scored from 1 (“very poor”) to 7 (“excellent”). All scales and single-item measures were linearly transformed to a scale of 0–100 using standard procedures (23). Higher functioning and global health status/quality of life scores indicated better function or health status; higher scores on symptom items/scales and financial impact indicated more symptom complaints and greater financial impact. Clinically meaningful differences in HRQL scores were determined using published guidelines (24,25).

#### Demographics and Clinical Data

The CAESAR questionnaire also contained questions concerning clinical history and sociodemographic factors. Self-reported comorbid conditions include stroke, myocardial infarction, angina pectoris, heart failure, arthrosis, rheumatism, osteoporosis, and diabetes mellitus. Participating cancer registries provided information on date of diagnosis and cancer stage. Information on treatment received and disease progression (recurrence or metastasis) was self-reported.

### Statistical Analyses

All analyses were conducted with SAS (version 9.4 for Windows; SAS Institute Inc., Cary, NC). We derived odds ratios (ORs) and 95% confidence intervals (CIs) of sociodemographic and clinical variables, health care use, and psychosocial distress associated with patient identity. All analyses were adjusted for age and sex, and cancer stage where appropriate. Although comorbidity differed between the groups, this variable was not included for adjustment as comorbidity reflected the situation at the time of the survey. It is therefore not considered a confounder as some of the differences associated with comorbidity could represent a consequence of the cancer.

Least square mean HRQL scores were calculated between survivors with a patient identity vs those who did not have a patient identity. Variables included for adjustment were age at survey, sex, and type of cancer. Two-sided statistical significance were determined at a *P* value of less than .05.

To reduce possible bias due to missing data (generally less than 10%), multiple imputation was conducted. Data were imputed with the Markov chain Monte Carlo method with 25 imputations.

### Sensitivity Analyses

We reran the analyses excluding survivors with advanced disease (stage IV) at the time of diagnosis and those who reported disease progression at the time of the survey.

## Results

### Survivors’ Characteristics

Of the 14_ _774 eligible participants, 6057 (41%) returned a complete questionnaire. Respondents were more likely to be male, less likely to have colorectal cancer, and younger at diagnosis (data not shown). There was no difference in cancer stage between respondents and nonrespondents.

Among the respondents, 25% of colorectal, 31% of breast, and 36% of prostate cancer survivors still perceived themselves as patients (Table 1). As results from using imputed data were comparable to those of nonimputed data (Table 1), we report all further results using imputed data. When compared with colorectal cancer survivors, prostate cancer survivors were more likely to perceive themselves as patients (OR_adj_ = 1.80, 95% CI = 1.49 to 2.18). Male survivors were more likely to have the patient identity when compared with female survivors (OR_adj_ = 1.37, 95% CI = 1.22 to 1.55). When stratified by gender and cancer type, prostate cancer survivors were more likely to have the patient identity (OR_adj_ = 1.79, 95% CI = 1.47 to 2.17) when compared with male colorectal cancer survivors. The odds of having a patient identity among breast cancer survivors when compared with female colorectal cancer survivors were reduced to trend statistical significance following adjustment. Younger age was also associated with the patient identity when compared with survivors older than age 80 years: 30 to 49 years (OR_adj_ = 1.70, 95% CI = 1.23 to 2.34), 50 to 59 years (OR_adj_ = 1.75, 95% CI = 1.38 to 2.23), 60 to 69 years (OR_adj_ = 1.25, 95% CI = 1.02 to 1.53). Having comorbid conditions increased the odds of having a patient identity: one condition (OR_adj_ = 1.33, 95% CI = 1.17 to 1.51), two or more conditions (OR_adj_ = 1.59, 95% CI = 1.38 to 1.83). Survivors with stage II cancer (OR_adj_ = 1.31, 95% CI = 1.15 to 1.51), stage III cancer (OR_adj_ = 1.61, 95% CI = 1.37 to 1.90), or stage IV cancer (OR_adj_ = 2.44, 95% CI = 1.83 to 3.25) at diagnosis were more likely to have a patient identity when compared with stage I survivors. Having disease recurrence increased the odds of having a patient identity (OR_adj_ = 4.08, 95% CI = 3.53 to 4.72), although this perception decreased with time since disease recurrence: less than two years (OR_adj_ = 3.74, 95% CI = 2.56 to 5.47), two to five years (OR_adj_ = 2.53, 95% CI = 1.87 to 3.42). Being in a partnered relationship, education level, and years since diagnosis were not associated with the patient identity.

**Table 1.** Description of study population and overall association between individual characteristics and proportion of survivors still perceiving themselves to be cancer patients

	Cancer survivors		Perceiving oneself still as cancer patient				
	No. (%col*)	MI %col	No. (%row)	MI %row	OR_crude_ (95% CI)	OR_adj_† (95% CI)	MI OR_adj_† (95% CI)
Total	6057 (100)		1902‖ (31)	33	–	–	–
Cancer type							
Colorectal	1217 (20)	20	299 (25)	26	1.00	1.00	1.00
Breast	2654 (44)	44	815 (31)	32	1.36 (1.16 to 1.59)	1.25 (1.00 to 1.57)	1.20 (0.97 to 1.50)
Prostate	2186 (36)	36	788 (36)	38	1.76 (1.50 to 2.06)	1.85 (1.52 to 2.24)	1.80 (1.49 to 2.18)
Sex							
Female	3158 (52)	52	937 (30)	31	1.00	1.00	1.00
Male	2899 (48)	48	965 (33)	35	1.20 (1.07 to 1.33)	1.38 (1.22 to 1.55)‡	1.37 (1.22 to 1.54)‡
Sex by cancer type							
Female							
CRC	504 (16)		122 (24)		1.00	1.00	1.00
Breast	2654 (84)		815 (31)		1.38 (1.10 to 1.72)	1.23 (0.98 to 1.54)‡	1.18 (0.94 to 1.47)‡
Male							
CRC	713 (25)		177 (25)		1.00	1.00	1.00
Prostate	2186 (75)		788 (36)		1.74 (1.43 to 2.10)	1.83 (1.50 to 2.22)‡	1.79 (1.47 to 2.17)‡
CRC							
Female	504 (41)		122 (24)		1.00	1.00	1.00
Male	713 (59)		177 (25)		1.03 (0.79 to 1.34)	1.04 (0.79 to 1.36)‡	1.02 (0.78 to 1.32)‡
Age at survey§, y							
30–49	242 (4)	4	88 (36)	38	1.39 (1.01 to 1.91)	1.69 (1.22 to 2.35)	1.70 (1.23 to 2.34)
50–59	683 (11)	11	262 (38)	39	1.50 (1.18 to 1.89)	1.77 (1.39 to 2.26)	1.75 (1.38 to 2.23)
60–69	1786 (29)	30	578 (32)	33	1.15 (0.94 to 1.41)	1.25 (1.02 to 1.54)	1.25 (1.02 to 1.53)
70–79	2734 (45)	45	797 (29)	31	1.01 (0.83 to 1.23)	1.03 (0.85 to 1.25)	1.04 (0.86 to 1.26)
80–89	611 (10)	10	176 (29)	30	1.00	1.00	1.00
Missing	1 (0.02)		1				
In a partnered relationship							
Yes	4754 (78)	80	1533 (32)	34	1.00	1.00	1.00
No	1212 (20)	20	340 (28)	30	0.82 (0.72 to 0.95)	0.92 (0.80 to 1.07)	0.92 (0.80 to 1.06)
Missing	91 (2)		29				
Education, y							
≤9	3162 (52)	53	968 (31)	32	1.00	1.00	1.00
10–11	1416 (23)	24	443 (31)	32	1.00 (0.87 to 1.15)	0.97 (0.84 to 1.11)	0.97 (0.85 to 1.11)
≥12	1359 (22)	23	457 (34)	35	1.13 (0.99 to 1.30)	1.03 (0.89 to 1.18)	1.03 (0.89 to 1.18)
Missing	120 (2)		34				
Comorbidities¶							
None	2683 (44)	44	763 (28)	30	1.00	1.00	1.00
1	1892 (31)	32	622 (33)	34	1.24 (1.09 to 1.41)	1.35 (1.18 to 1.54)	1.33 (1.17 to 1.51)
≥2	1451 (24)	24	510 (35)	37	1.39 (1.21 to 1.60)	1.61 (1.40 to 1.68)	1.59 (1.38 to 1.83)
Missing	31 (1)		7				
Years since diagnosis							
5–7	2855 (47)	47	947 (33)	35	1.00	1.00	1.00
8–9	1969 (33)	33	591 (30)	31	0.85 (0.75 to 0.97)	0.88 (0.78 to 1.00)	0.88 (0.78 to 0.99)
≥10	1192 (20)	20	357 (30)	31	0.85 (0.74 to 0.99)	0.91 (0.78 to 1.06)	0.92 (0.79 to 1.06)
Missing	41 (1)		7				
Stage at diagnosis							
I	1446 (24)	28	374 (26)	27	1.00	1.00	1.00
II	2266 (37)	47	712 (31)	33	1.35 (1.16 to 1.57)	1.36 (1.16 to 1.59)	1.31 (1.15 to 1.51)
III	996 (16)	21	354 (36)	38	1.59 (1.33 to 1.89)	1.62 (1.34 to 1.96)	1.61 (1.37 to 1.90)
IV	187 (3)	4	85 (45)	48	2.51 (1.83 to 3.45)	2.56 (1.85 to 3.55)	2.44 (1.83 to 3.25)
Missing	1162 (19)		377				
Disease recurrence							
No	5036 (83)	84	1320 (26)	28	1.00	1.00	1.00
Yes (any)	934 (15)	16	556 (60)	61	4.23 (3.65 to 4.91)	4.19 (3.61 to 4.86)	4.08 (3.53 to 4.72)
Missing	87 (1)		26				
If yes, time since recurrence, y							
<2	201 (22)	22	148 (74)	76	3.68 (2.48 to 5.46)	3.74 (2.51 to 5.58)	3.74 (2.56 to 5.47)
2–5	321 (34)	39	215 (67)	68	2.67 (1.92 to 3.71)	2.71 (1.94 to 3.78)	2.53 (1.87 to 3.42)
≥6	314 (34)	39	140 (45)	46	1.00	1.00	1.00
Missing	98 (10)		53				

* %column might not add up to 100% due to rounding up of decimals. CI = confidence interval; CRC = colorectal cancer; MI = multiple imputation, based on 25 imputations; OR = odds ratio.

† Adjusted for sex and age at survey, unless otherwise stated.

‡ Adjusted for age at survey.

§ Adjusted for sex.

‖ Respondents were missing information on patient identity (n = 226, 4%).

¶ Self-reported comorbid conditions include stroke, myocardial infarction, angina pectoris, heart failure, arthrosis, rheumatism, osteoporosis, and diabetes mellitus.

### Associations With Treatment

In general, treatment was associated with the patient identity, except among female colorectal cancer survivors (Table 2). Breast cancer survivors who had breast-preserving surgery were less likely to have the patient identity (OR_adj_ = 0.62, 95% CI = 0.51 to 0.75) than survivors who received mastectomy. Chemotherapy treatment was associated with increased odds of having a patient identity, but only among male colorectal (OR_adj_ = 1.78, 95% CI = 1.20 to 2.63) and prostate (OR_adj_ = 1.95, 95% CI = 1.47 to 2.58) cancer survivors. Male colorectal (OR_adj_ = 1.56, 95% CI = 1.06 to 2.30) and prostate cancer (OR_adj_ = 1.97, 95% CI = 1.65 to 2.36) survivors treated with radiotherapy were more likely to still consider themselves patients. Among breast and prostate cancer survivors, receiving hormone therapy was associated with higher odds of having a patient identity (OR_adj_ = 1.31, 95% CI = 1.11 to 1.54, and OR_adj_ = 4.27, 95% CI = 3.60 to 4.40, respectively).

**Table 2.** Cancer-specific association between treatment characteristics and proportions of survivors still perceiving themselves as cancer patients (after multiple imputation of missing values)

	Breast cancer (female)			Colorectal cancer (female)			Colorectal cancer (male)			Prostate cancer (male)		
	Total No.	Still cancer patient		Total No.	Still cancer patient			Still cancer patient			Still cancer patient	
		No. (%)	OR_adj_.* (95% CI)		No. (%)	OR_adj._* (95% CI)	Total No.	No. (%)	OR_adj._* (95% CI)	Total No.	No. (%)	OR_adj._* (95% CI)
Overall	2654	847 (32)	–	504	132 (26)	–	713	187 (26)	–	2186	823 (38)	–
Organ-preserving therapy†												
No	638	259 (41)	1.00	48	12 (24)	1.00	96	31 (33)	1.00	518	115 (22)	1.00
Yes	2016	588 (29)	0.62 (0.51 to 0.75)	456	121 (26)	1.23 (0.60 to 2.50)	617	156 (25)	0.73 (0.46 to 1.18)	176	40 (23)	1.09 (0.72 to 1.65)
Chemotherapy												
No	1055	308 (29)	1.00	267	63 (21)	1.00	390	81 (21)	1.00	1961	703 (36)	1.00
Yes	1599	539 (34)	0.96 (0.79 to 1.16)	237	69 (28)	1.23 (0.78 to 1.93)	323	106 (33)	1.78 (1.20 to 2.63)	224	119 (53)	1.95 (1.47 to 2.58)
Radiation												
No	437	145 (33)	1.00	370	94 (25)	1.00	537	128 (24)	1.00	1258	387 (31)	1.00
Yes	2217	702 (32)	0.92 (0.74 to 1.15)	134	37 (29)	1.11 (0.70 to 1.76)	176	60 (34)	1.56 (1.06 to 2.30)	928	436 (47)	1.97 (1.65 to 2.36)
Hormone therapy												
No	1363	392 (29)	1.00	–	–	–	–	–	–	1593	465 (29)	1.00
Yes	1291	455 (35)	1.31 (1.11 to 1.54)	–	–	–	–	–	–	593	358 (60)	3.60 (2.94 to 4.40)

* Adjusted for age at survey and cancer stage. CI = confidence interval; OR = odds ratio.

† Organ preserving therapy: a) breast cancer: yes = breast conservation (including breast reconstruction following mastectomy), no = mastectomy only; b) colorectal cancer: yes = no stoma, no = has permanent stoma. For prostate cancer, this was limited to stage I–II respondents (n = 594) treated either with or without surgery only: yes = no prostatectomy, no = prostatectomy.

### Associations With Aspects of Care and Health Care Use

Survivors who reported still receiving cancer treatment or aftercare were more likely (OR_adj_ = 13.34, 95% CI = 11.66 to 15.26) to still consider themselves patients when compared with survivors who reported that their treatment or aftercare had been completed (Table 3).

**Table 3.** Cross-sectional association between “perceiving oneself still as cancer patient” and aspects of care (after multiple imputation of missing values)

	Cancer survivors	Perceiving oneself still as cancer patient		
	No. (%column*)	No.† (%row)	OR_crude_ (95% CI)	OR_adjusted_ (95% CI)‡
Total	6057 (100)	1990 (33)	–	–
Cancer aftercare or treatment has terminated				
Yes	4294 (71)	719 (17)	1.00	1.00
No	1763 (29)	1271 (72)	12.82 (11.26 to 14.64)	13.34 (11.66 to 15.26)
Cancer-related health care use during past 12 mo				
Consulted a:				
General practitioner				
Yes	2066 (34)	928 (45)	2.25 (2.02 to 2.52)	2.24 (2.00 to 2.50)
No	3991 (66)	1061 (27)	1.00	1.00
Medical specialist (oncologist, psychologist)				
Yes	4395 (73)	1698 (39)	2.96 (2.57 to 3.40)	2.89 (2.51 to 3.33)
No	1662 (27)	292 (18)	1.00	1.00
Nonmedical practitioner (CAM practitioner)				
Yes	167 (3)	79 (47)	1.86 (1.36 to 2.53)	1.80 (1.32 to 2.45)
No	5890 (97)	1911 (32)	1.00	1.00
Received treatment at:				
Hospital care (acute care)				
Yes	328 (5)	198 (60)	3.35 (2.67 to 4.21)	3.36 (2.67 to 4.23)
No	5729 (95)	1791 (31)	1.00	1.00
University hospital				
Yes	85 (1)	51 (60)	3.17 (2.04 to 4.90)	3.05 (1.96 to 4.73)
No	5972 (99)	1938 (32)	1.00	1.00
Rehabilitation hospital				
Yes	163 (3)	87 (53)	2.41 (1.76 to 3.30)	2.36 (1.72 to 3.23)
No	5894 (97)	1903 (32)	1.00	1.00

* %column might not add up to 100% due to rounding off of decimals. CAM = complementary/alternative medicine; CI = confidence interval; OR = odds ratio.

† No. might not add up to the total of 1990 due to rounding off of decimals.

‡ Adjusted for sex and age at survey.

Patient identity was associated with cancer-related health care use in the past 12 months, with higher odds for visits to the GP (OR_adj_ = 2.24, 95% CI = 2.00 to 2.50), MS (OR_adj_ = 2.89, 95% CI = 2.51 to 3.33), and NP (OR_adj_ = 1.80, 95% CI = 1.32 to 2.45). Survivors who still perceived themselves as patients were also more likely to have received care in the AH (OR_adj_ = 3.36, 95% CI = 2.67 to 4.23), UH (OR_adj_ = 3.05, 95% CI = 1.96 to 4.73), or RH (OR_adj_ = 2.36, 95% CI = 1.72 to 3.23) (Table 4).

**Table 4.** Cross-sectional association between “perceiving oneself still as cancer patient” and psychosocial distress (after multiple imputation of missing values)

	Cancer survivors	Perceiving oneself still as cancer patient		
	No.* (% column†)	No.* (% row)	OR_crude_ (95% CI)	MI OR_adjusted_ (95% CI)‡
Total	6057 (100)	1990 (33)		
How much strain are you currently experiencing from cancer?				
Very much	433 (7)	372 (86)	55.24 (40.97 to 74.48)	55.96 (41.44 to 75.57)
Moderate	882 (15)	567 (64)	16.24 (13.48 to 19.57)	16.54 (13.70 to 19.97)
Low	2042 (34)	781 (38)	5.59 (4.79 to 6.52)	5.74 (4.90 to 6.71)
None	2700 (45)	269 (10)	1.00	1.00
Cancer-related distress (QSC-R10; range = 0–50 points)				
Yes (>14 points)	1991 (33)	945 (47)	2.62 (2.34 to 2.93)	2.62 (2.33 to 2.93)
No ( 0–14 points)	4066 (67)	1044 (26)	1.00	1.00
Depression (GDS; range = 0–15 points)				
Depressed (11–15 points)	270 (4)	156 (58)	3.46 (2.70 to 4.44)	3.47 (2.70 to 4.47)
Subclinical depression (5–10 points)	1223 (20)	541 (44)	2.01 (1.77 to 2.29)	2.05 (1.80 to 2.34)
No (0–4 points)	4564 (75)	1292 (28)	1.00	1.00
Fear of recurrence (FoP-Q-SF)				
High	240 (4)	157 (66)	4.61 (3.51 to 6.05)	4.83 (3.66 to 6.37)
Moderate	540 (9)	291 (54)	2.82 (2.36 to 3.38)	2.99 (2.49 to 3.59)
Mild	5276 (87)	1541 (29)	1.00	1.00

* No. might not add up to the total of 6057 or 1990 due to rounding off of decimals. CI = confidence interval; FoP-Q-SF = Fear of Progression Questionnaire-Short Form (high: at least 75% of items have a score of ≥4; moderate: at least 50% of items have a score of ≥4); GDS = Geriatric Depression Scale; OR = odds ratio; QSC-R10 = Questionnaire on Stress in Cancer Patients.

† %column might not add up to 100% due to rounding up of decimals.

‡ Adjusted for sex and age at survey.

### Associations With Psychosocial Distress

Survivors who reported currently experiencing strain from cancer were more likely to have a patient identity: little (OR_adj_ = 5.74, 95% CI = 4.90 to 6.71), moderate (OR_adj_ = 16.54, 95% CI = 13.70 to 19.97), and very much (OR_adj_ = 55.96, 95% CI = 41.44 to 75.57) (Table 3). Similarly, survivors with a patient identity were more likely to report higher levels of cancer-related distress (OR_adj_ = 2.62, 95% CI = 2.33 to 2.93) and were more likely to be depressed: subclinical (OR_adj_ = 2.05, 95% CI = 1.80 to 2.34) and clinical (OR_adj_ = 3.47, 95% CI = 2.70 to 4.47). Furthermore, having a moderate (OR_adj_ = 2.99, 95% CI = 2.49 to 3.59) to high (OR_adj_ = 4.83, 95% CI = 3.66 to 6.37) fear of disease recurrence was associated with the patient identity.

### Associations With HRQL

**Figure 1.**
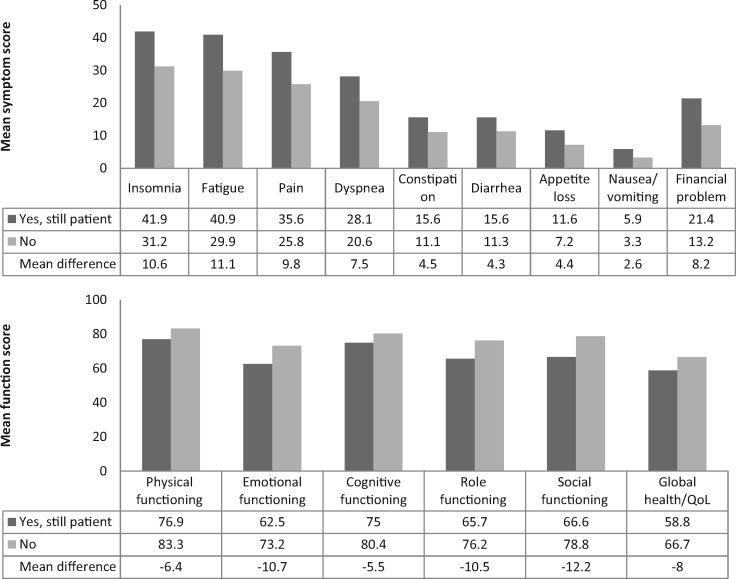
Mean European Organization for Research and Treatment of Cancer (EORTC) scale scores by status of “perceiving oneself still as cancer patient,” after imputation of missing values. Means are adjusted for age at survey and sex. EORTC Quality of Life Core-30: higher scores indicated better function or health status but more symptom complaints or financial problems. All scores were significantly different at a *P* value of less than .0001. Differences in the mean difference score could be due to rounding up of decimals. QoL = quality of life.

Survivors who identified themselves as still being patients reported statistical significantly lower scores on all the functioning and global health/quality of life subscales, and higher symptom burden and financial difficulties (Figure 1) when compared with survivors who did not perceive themselves as still being patients. Most of these differences were of trivial or small clinical significance, except for differences in the emotional and social functioning subscales, which were of medium clinical significance (24,25).

### Sensitivity Analyses

Excluding respondents with advanced disease or those who had disease progression before the survey showed similar results, albeit generally with reduced odds ratios. Within this group of stage I–III disease-free respondents, having a patient identity was associated with higher health care use, greater psychosocial distress, and lower HRQL (Supplementary Tables 1 and 2 and Supplementary Figure 1, available online). Of note, the association between patient identity and a high fear of recurrence was increased among disease-free survivors (Supplementary Table 2, available online).

## Discussion

This population-based study of (very) long-term survivors of breast, colorectal, and prostate cancer found that a significant proportion of survivors still perceive themselves as patients 5 to 15 years after cancer diagnosis. Prevalence estimates of survivors who still consider themselves patients found in this study were within the range reported for breast and prostate cancers but higher for colorectal cancer, when compared with previous studies (8). In our study, breast and prostate cancer survivors were more likely to identify themselves as patients when compared with colorectal cancer survivors. Similarly, a previous study reported that long-term prostate cancer survivors were less likely to identify as survivors when compared with colorectal cancer survivors (5). It is possible that treatment could contribute to this perception, as prostate cancer survivors receiving hormone treatment were aware that such treatment is not curative and is of long duration (26).

Breast cancer survivors who did not have organ-preserving treatments (ie, had mastectomies) were more likely to perceive themselves as still being patients. In a qualitative study, breast cancer survivors described the loss of breast as having a significant negative impact on perceptions of their femininity and relationships (27). In contrast, a study has also reported the positive effect of mastectomy, where breast removal was seen as removing the cancer from the body (28). Similarly, prostate cancer survivors treated surgically were less likely to have a patient identity. However, low-risk stage I–II prostate cancer survivors who received organ-preserving treatment (no prostatectomy) were more likely to have a patient identity, although results were not significant, probably due to the small numbers. It is possible that living with untreated cancer (eg, managed with active surveillance) could have negative psychological consequences (29).

In our study, younger survivors were more likely to have a patient identity when compared with elderly survivors. Similarly, a study of long-term colorectal cancer survivors reported that younger survivors were less likely to endorse the survivor identity when compared with older survivors (10). This could be due to the normalization process whereby older individuals perceive cancer as a chronic illness that could be expected as part of the life course or to older individuals having experience living with other precancer chronic conditions (“normal hardship theory”), thus lessening the impact of cancer in their lives (30,31). We found that having at least one comorbid condition increased the odds of having a patient identity. Older individuals could also consider the symptoms and side effects of cancer and its treatment to be symptomatic of the aging process (32); for example, older males without prostate cancer could also have complaints of urinary incontinence and sexual dysfunction (33).

Results from this study suggest a degree of interconnectedness. Experiencing a disease recurrence or metastasis increased the odds of having a patient identity, although this perception was strongest within two years of the event and reduced with time. Survivors who reported still receiving cancer treatment or aftercare strongly associated themselves with the patient identity. More than 60% of survivors who reported currently experiencing moderate to very much strain from cancer endorsed the patient identity. These survivors were also more likely to have had a disease recurrence (data not shown). Previously, we found that long-term cancer survivors who experienced disease progression had poorer psychological well-being when compared with disease-free long-term survivors (34). After excluding survivors with advanced disease or disease progression, survivors who reported to be still receiving treatment/aftercare or feeling strain from cancer were still associated with significantly higher odds of having a patient identity. Breast cancer or prostate cancer survivors who reported being treated with hormone therapy were also more likely to endorse the patient identity. In turn, continued maintenance hormone therapy could contribute to feeling strain from cancer, which was also associated with significantly higher odds of having a patient identity. Other factors such as personality or illness perceptions could also play a role in developing/maintaining a patient identity (35). In the current study, cancer-related distress and meeting subclinical/clinical indicators of depression were associated with an increased vulnerability to having a patient identity. Furthermore, survivors with a patient identity were more likely to have made cancer-related visits to health care specialists or facilities in the past 12 months. We could not find published results on the association between patient identity and health care use for comparison, although a study of long-term endometrial cancer survivors showed that cancer worry was associated with higher health care use (36). Taken together, these results are intuitive, as the continued reminder of cancer can maintain the patient identity. Future studies could look into mediating relationships between the factors identified in this study that are associated with the patient identity.

We found that survivors with a moderate to high fear of disease recurrence were more likely to have a patient identity. This result is congruent with those of previous studies, that individuals ascribing to the patient identity also have more concerns about cancer recurrence (8).

The current results highlight the importance of considering illness identity when planning cancer interventions (37). Research suggests that a patient identity might reduce feelings of control and diminish the individual’s role in shared treatment decision-making (38). In a qualitative study, survivors’ illness identities were found to influence the decision of whether to participate in a clinical trial (39). On the other hand, the push toward the concept of survivorship has been critiqued as encouraging cancer patients to conceal their physical symptoms and stigmatizing their feelings of suffering (14).

This study has limitations. Cancer identity was assessed with a dichotomized forced choice question. Qualitative studies have shown that cancer identity can be a complex and fluid construct, which might not be adequately assessed in this study (40). The cross-sectional design does not allow causal associations of cancer identity to be established. For example, we found associations of patient identity with hormone therapy, and also with health care use. But hormone therapy was also associated with health care use (data not shown). Therefore, it is not possible to establish the direction of this relationship between patient identity and health care use. Furthermore, patient-reported outcomes, clinical information, and health care use data were self-reported, raising the possibility of recall bias. Confidence limits were not adjusted for multiple testing, so they refer to individual rather than simultaneous comparisons. Nevertheless, the strengths of this quantitative study include the large population-based sample of long-term and very long-term survivors who provided data on an extensive range of demographic, clinical, health care use, and psychosocial factors.

In conclusion, a significant proportion of (very) long-term cancer survivors still consider themselves patients. The patient identity is associated with a wide range of demographic, clinical, and psychosocial factors.

## Funding

This work was supported by a grant from the German Cancer Aid (No. 108262). The funding source was not involved in the collection, interpretation, or analysis of the data; in the writing of the manuscript; or in the decision to submit this report for publication.

## Notes

Affiliations of authors: Unit of Cancer Survivorship, Division of Clinical Epidemiology and Aging Research, German Cancer Research Center (DKFZ), Heidelberg, Germany (MSYT, VA); Institute for Social Medicine and Epidemiology, University of Lübeck, Lübeck, Germany (EMW, AW); Division of Clinical Epidemiology and Aging Research, DKFZ, Heidelberg, Germany (LKG, HB); Hamburg Cancer Registry, Ministry of Health and Consumer Protection, Hamburg, Germany (AW, MWS); Cancer Registry of Schleswig-Holstein, Lübeck, Germany (RP); Cancer Registry of North Rhine-Westphalia, Bochum, Germany (HB, HK); Bremen Cancer Registry, Leibniz Institute for Prevention Research and Epidemiology - BIPS, Bremen, Germany (AE); Saarland Cancer Registry, Saarbrücken, Germany (BH); Cancer Registry of Rhineland-Palatinate, Mainz, Germany (SRZ); Division of Preventive Oncology, DKFZ and National Center for Tumor Diseases (NCT), Heidelberg, Germany (HB); German Cancer Consortium (DKTK), DKFZ, Heidelberg, Germany (HB).

EMW has declared a consulting role, and AW has declared receiving honoria for teaching lectures; these roles do not bias the work or interfere with objective judgment of this manuscript. The rest of the co-authors have no conflicts of interest.

Data from this article have been presented at the following meetings: German Society for Epidemiology Annual Meeting, Lübeck 2017; International Association of Cancer Registries Meeting, Utrecht 2017; DKFZ Conference on Preventive Oncology, Heidelberg 2018.

## Supplementary Material

Supplementary Data
