# Supplementary material for: “Still a Cancer Patient”—Associations of Cancer Identity With Patient-Reported Outcomes and Health Care Use Among Cancer Survivors
Source: JNCI Cancer Spectr. 2018 Jul 5;2(2):pky031. doi: 10.1093/jncics/pky031 (PMC6649846; doi:10.1093/jncics/pky031)
Supplement: Supplementary Data [file pky031_still_cancer_patient_supp_tables_jncics_rev1_proof.docx]

**Supplementary Tables and Figure - CLEAN COPY**

**Supplementary Table 1: Cross-sectional association between “perceiving oneself still as cancer patient” and aspects of care, after multiple imputation of missing values. Stage I-III disease-free respondents**

|  | **Cancer Survivors** | |  | **Perceiving oneself still as cancer patient** | | | | | |
| --- | --- | --- | --- | --- | --- | --- | --- | --- | --- |
|  | ***N**** | **%column**** |  | ***N**** | | **%row** | | **OR _crude_ (95% CI)** | **OR _adj_. (95% CI)***** |
| **Total** | 4948 | 100 |  | 1348 | 27 | | - | | - |
| **Cancer aftercare or treatment has terminated** |  |  |  |  |  | |  | |  |
| Yes | 3840 | 78 |  | 608 | 16 | | 1.00 | | 1.00 |
| No | 1109 | 22 |  | 739 | 67 | | 10.63 (9.13-12.37) | | 11.02 (9.43-12.86) |
|  |  |  |  |  |  | |  | |  |
| **Cancer-related health care use during past 12 months** |  |  |  |  |  | |  | |  |
| *Consulted a:* |  |  |  |  |  | |  | |  |
| **General practitioner** |  |  |  |  |  | |  | |  |
| Yes | 1514 | 31 |  | 562 | 37 | | 1.99 (1.74-2.26) | | 1.98 (1.74-2.26) |
| No | 3434 | 69 |  | 786 | 23 | | 1.00 | | 1.00 |
| **Medical specialist (oncologist, psychologist)** |  |  |  |  |  | |  | |  |
| Yes | 3457 | 70 |  | 1109 | 32 | | 2.48 (2.12-2.90) | | 2.45 (2.09-2.86) |
| No | 1491 | 30 |  | 239 | 16 | | 1.00 | | 1.00 |
| **Non-medical practitioner (CAM practitioner)** | | |  |  |  | |  | |  |
| Yes | 112 | 2 |  | 44 | 39 | | 1.75 (1.19-2.56) | | 1.65 (1.12-2.43) |
| No | 4836 | 98 |  | 1304 | 27 | | 1.00 | | 1.00 |
| *Received treatment at:* |  |  |  |  |  | |  | |  |
| **Hospital (acute care)** |  |  |  |  |  | |  | |  |
| Yes | 155 | 3 |  | 71 | 46 | | 2.31 (1.67-3.19) | | 2.31 (1.67-3.20) |
| No | 4794 | 97 |  | 1277 | 27 | | 1.00 | | 1.00 |
| **University hospital** |  |  |  |  |  | |  | |  |
| Yes | 38 | 1 |  | 16 | 42 | | 1.97 (1.03-3.76) | | 1.96 (1.02-3.76) |
| No | 4911 | 99 |  | 1332 | 27 | | 1.00 | | 1.00 |
| **Rehabilitation hospital** |  |  |  |  |  | |  | |  |
| Yes | 88 | 2 |  | 36 | 41 | | 1.91 (1.24-2.94) | | 1.88 (1.22-2.89) |
| No | 4860 | 98 |  | 1311 | 27 | | 1.00 | | 1.00 |

OR: odds ratio; 95% CI: 95% confidence interval

* *N* might not add up to the total of 4948 or 1348 due to rounding off of decimal

** %column might not add up to 100% due to rounding off of decimal

*** adjusted for sex and age at survey

CAM: complementary/alternative medicine

**Supplementary Table 2: Cross-sectional association between “perceiving oneself still as cancer patient” and psychosocial distress, after multiple imputation of missing values. Stage I-III disease-free respondents**

|  | **Cancer Survivors** | |  | **Perceiving oneself still as cancer patient** | | | |
| --- | --- | --- | --- | --- | --- | --- | --- |
|  | ***N**** | **% column*** |  | ***N*** | **% row** | **OR_crude_ (95% CI)** | **OR_adj._(95% CI)***** |
| **Total** | 4948 | 100 |  | 1348 | 27 |  |  |
| **How much strain are you currently experiencing from the cancer?** |  |  |  |  |  |  |  |
| Very much | 215 | 4 |  | 176 | 82 | 48.96 (33.63-71.28) | 49.08 (33.67-71.55) |
| Moderate | 636 | 13 |  | 373 | 59 | 15.25 (12.33-18.87) | 15.42 (12.44-19.12) |
| Low | 1684 | 34 |  | 594 | 35 | 5.88 (4.94-7.01) | 5.94 (4.97-7.09) |
| None | 2413 | 49 |  | 205 | 8 | 1.00 | 1.00 |
| **Cancer-related distress (QSC-R10). Range: 0-50 points** | |  |  |  |  |  |  |
| Yes (>14 points) | 1500 | 30 |  | 604 | 40 | 2.45 (2.15-2.79) | 2.43 (2.13-2.77) |
| No ( 0-14points) | 3449 | 70 |  | 744 | 22 | 1.00 | 1.00 |
| **Depression (GDS). Range 0-15 points** |  |  |  |  |  |  |  |
| Depressed (11-15 points) | 190 | 4 |  | 99 | 52 | 3.54 (2.64-4.76) | 3.54 (2.63-4.76) |
| Subclinical depression (5-10 points) | 934 | 19 |  | 346 | 37 | 1.90 (1.63-2.22) | 1.93 (1.65-2.25) |
| No (0-4 points) | 3825 | 77 |  | 903 | 24 | 1.00 | 1.00 |
| **Fear of recurrence (FoP-Q-SF)** |  |  |  |  |  |  |  |
| High | 170 | 3 |  | 106 | 63 | 5.22 (3.80-7.17) | 5.31 (3.84-7.32) |
| Moderate | 390 | 8 |  | 179 | 46 | 2.65 (2.14-3.27) | 2.74 (2.21-3.39) |
| Mild | 4388 | 89 |  | 1063 | 24 | 1.00 | 1.00 |

OR: odds ratio; 95% CI: 95% confidence interval

* *N* might not add up to the total of 4948 or 1348 due to rounding off of decimal

* %column might not add up to 100% due to rounding up of decimal

** adjusted for sex and age at survey

QSC-R10: Questionnaire on Stress in Cancer Patients

GDS: Geriatric Depression Scale

FoP-Q-SF: Fear of Progression Questionnaire-Short Form. High: at least 75% items have score of ≥4; Moderate: at least 50% items have a score of ≥4

**Supplementary Figure 1: Mean EORTC scale scores by status of “perceiving oneself still as cancer patient” after multiple imputation of missing values. Stage I-III disease-free respondents**

Means are adjusted for age at survey and sex

EORTC-QLQ-C30: higher scores indicated better function or health status but more symptom complaints or financial problems. All scores were significantly different at p<0.0001. Differences in the mean difference score could be due to rounding up of decimal.
